# Ophthalmology

**Published:** 1905-05-13

**Authors:** 


### OPHTHALMOLOGY.

**Amblyopia ex Anopsia.**—St. John Roosa [1] contributes a paper on this subject which is of great value owing to the accurate observations that were possible. The patient was a man, 46 years of age. He had had convergent strabismus from early childhood in his right eye. An operation was done which over-corrected the defect and left him with divergent strabismus. This was eventually corrected. The eye showed a hypermetropic astigmatism of 4 dioptres. The vision of the eye was 5-60 without a glass, and with a +4 cylinder 5-36 or thereabout, and could not be improved further. His left eye, on which was his only dependence for vision, then had an accident which ruptured the cornea and set up traumatic cataract. This latter was operated on, but the resulting vision in that eye was only 5-60 with a +11 sphere. Dr. Roosa then turned his attention to the amblyopic right eye, correcting the astigmatism with +4 cyl. and adding +4 spherical for reading. At first the patient could not read ordinary type, but was advised to exercise his eyes in reading, beginning with children's primers, hope being held out to him that the vision would ultimately improve. One and a half years later his distant vision was not in the least improved, but he was able to read No. 1 Jaeger with his glass. Eighteen months later he appeared with his distant vision equal to 5-9 with an appropriate glass, his corneal astigmatism reduced from 5 to 4 dioptres. The fixation in the right eye was now central and steady. Vision in the injured left eye remained as before, at 5-60. The interest in the case, Dr. Roosa says, is twofold. First, that a man more than 40 years old, after having had an amblyopic eye ever since his early youth, could develop his eyesight to very nearly normal visual power. Secondly, that the vision for fine type was much improved some months before that for letters at 20 feet was improved at all. The case also shows in a most interesting way not only the possibility of relief in such cases, where the good eye is so much injured as to cause a great impairment of sight, but also it demonstrates the practicability and importance of the treatment first thoroughly carried out by Javal, and widely taught by him, of exercise of an amblyopic eye for the purpose of removing what turns out to be functional amblyopia.

**Wound of Eyeball from Christmas Cracker.**—Leslie Buchanan[2] mentions a case of some interest, though perhaps not very urgent at this season of the year, still of great moment to parents when purchasing their next Christmas supply of crackers. The patient, a girl of 19, said that she had received a blow on the right eye while pulling a cracker the previous evening—there was a large wound at the ciliary region—it was radially situated, very ragged, and allowed of prolapse of both iris and ciliary body. As it was suspected from the appearance of the wound that there was a foreign body in the eye, the point of a large electro-magnet was brought close to it, and at once a large shining body came to the surface and was easily seen through both the pupil and the wound. A small magnet was then used, but though the metal rose to it well it could not be induced to come through the wound owing to its large size. Ultimately, after considerable enlargement of the wound, a piece of tinplate, measuring a quarter of an inch square, much curved, and with very ragged edges, was removed from the eye. Contrary to expectation the eye did well under atropine with "cold treatment." The subsequent history of the case was that of a very successful cataract extraction. Several points of interest crop up in this case. When the magnet was tried it was only with faint hopes of success; so far as was known there should be no metal in the composition of a cracker. The fact that in some of these crackers there is metal shows that they are not such safe things to put into the hands of children as was formerly thought. The explosive part consists generally of two strips of paper coated probably with chlorate of potassium and a little sand, and it is by drawing these strips quickly past one another that the explosion is produced. Formerly the strips were held in apposition by a binding strip of paper, but in the cracker used in this case the strips were held together by a piece of tinplate lapped round them in such a manner that the explosion took place inside the fold of tin. In one of the crackers fired as an experiment the tin was burst open so violently that a portion of it passed out through the paper wrappings, and went eight feet across a room. The name of the maker of these crackers could not be obtained.

**Treatment of Glaucoma in Emergencies.**—The other day we were asked by a country practitioner what he should do in a case of acute glaucoma where eserine failed to lower the tension and no expert help was at hand. In such emergency we see Maitland Ramsay [3] recommends whenever the practitioner satisfies himself that the case is one of glaucoma he ought at once to instil eserine, put leeches to the temples, and apply hot fomentations to the eye. If in a few hours the pain has not subsided means must be taken to reduce the intraocular tension by opening the eyeball, so as to relieve the strangulation at the corneo-iridic angle. If the practitioner cannot obtain assistance he ought not to wait, but should at once puncture the sclerotic with a narrow knife, and allow some of the vitreous to escape. If he observes strict antiseptic precautions this can be done without danger, and will give relief till the services of a specialist can be obtained. The all-important thing is that the case be taken in time. While on this subject it is interesting to note Grandclement's[4] experience with adrenalin in the treatment of glaucoma. The case which first raised in his mind the idea that adrenalin might be of use in glaucoma was that of a young man who was not suffering from glaucoma at all but from a hyperæmia of the conjunctiva. The drug was given solely for its power to reduce hyperæmia. After a time the patient began to complain of failure of sight in the eye, and on examination it was discovered that the tension of the eye was very low. The use of the drug was at once discontinued, but it was fully six months before vision returned to its original standard, and the tension had risen to normal. He next tried it on a patient a woman of 37, who exhibited all the classical features of subacute glaucoma, following on a series of premonitory attacks. Before trying adrenalin he used eserine alone for 36 hours vigorously, but without the smallest result. He then instilled drops containing eserine with adrenalin every half hour. In two hours pain was lessened, in 10 hours hand movements could be perceived, in 24 hours patient could guide herself with the affected eye alone, on the third day the tension was normal, and in a week vision was two-thirds of normal. On a tendency to recurrence it was thought better to continue the drug in diminished quantities for some weeks. For a month there were tendencies to recurrence, but the high standard of vision remained, at least for some months. Grandclement, therefore, urges that in all suitable cases, *e.g.* where the patient refuses absolutely to have an iridectomy performed this method of treatment should be tried. He does not, however, consider that old standing cases should come within this category. Even in the less classical forms of glaucoma he advocates the use of adrenalin and eserine combined; he has employed them successfully in secondary glaucoma and buphthalmos. He lays down four axioms in regard to the value of these drugs. 1. That the glaucoma must be recent, there must be no organic changes in the tissues and particularly in the parts about the corneo-iridic angle. 2. That the adrenalin must be instilled every half hour without any intermission whatever, for, perhaps, several days, until the tension has gone down. 3. That eserine and adrenalin must be used together; the former acting by keeping the pupil small and the corneo-iridic angle open, the latter by diminishing the hyperæmia and hypersecretion of the ciliary body, thus combating both the principal elements in the causation of glaucoma. 4. That the employment of adrenalin must be stopped or at least diminished as soon as the tension becomes lowered to normal, lest a permanent condition of low tension should be established. The action of adrenalin though transitory is powerful, and it is capable, if employed too long, of causing a disease of the arteries resembling atheroma; indeed one of the current theories of the causation of atheroma is that it is brought on by an abnormal activity of the suprarenal glands.

[1] Med. Rec., Feb. 25, 1905. [2] Lancet, Jan. 14, 1905. [3] Ibid., [4] Edin. Med. Jour., December, 1904.

